# Impact and relationship of anterior commissure and time-dose factor on the local control of T1N0 glottic cancer treated by 6 MV photons

**DOI:** 10.1186/1748-717X-6-53

**Published:** 2011-05-21

**Authors:** Chi-Chung Tong, Kwok-Hung Au, Roger KC Ngan, Sin-Ming Chow, Foon-Yiu Cheung, Yiu-Tung Fu, Joseph SK Au, Stephen CK Law

**Affiliations:** 1 Department of Clinical Oncology, Queen Elizabeth Hospital, 30 Gascoigne Road, Kowloon, Hong Kong

**Keywords:** T1N0 glottic cancer, radiotherapy, 6 MV, anterior commissure, Biologically effective dose

## Abstract

**Background:**

To evaluate prognostic factors that may influence local control (LC) of T1N0 glottic cancer treated by primary radiotherapy (RT) with 6 MV photons.

**Methods:**

We retrospectively reviewed the medical records of 433 consecutive patients with T1N0 glottic cancer treated between 1983 and 2005 by RT in our institution. All patients were treated with 6 MV photons. One hundred and seventy seven (41%) patients received 52.5 Gy in 23 fractions with 2.5 Gy/fraction, and 256 (59%) patients received 66 Gy in 33 fractions with 2 Gy/fraction.

**Results:**

The median follow-up time was 10.5 years. The 10-year LC rates were 91% and 87% for T1a and T1b respectively. Multivariate analysis showed LC rate was adversely affected by poorly differentiated histology (Hazard Ratio [HR]: 7.5, *p *= 0.035); involvement of anterior commissure (HR: 2.34, *p *= 0.011); fraction size of 2.0 Gy (HR: 2.17, *p *= 0.035) and tumor biologically effective dose (BED) < 65 Gy_15 _(HR: 3.38, *p *= 0.017).

**Conclusions:**

The negative impact of anterior commissure involvement could be overcome by delivering a higher tumor BED through using fraction size of > 2.0 Gy. We recommend that fraction size > 2.0 Gy should be utilized, for radiation schedules with five daily fractions each week.

## Background

Laryngeal cancer is the third most common head and neck (H&N) cancer in Hong Kong. The age-standardized incidence rate was 2.3 per 100,000 [1] and is comparable to those of other developed countries like USA, the Netherlands and Japan. In Hong Kong, around 95% of early glottic cancer (GC) patients were treated by primary radiotherapy (RT) alone [2].

There is extensive published data regarding management of early GC treated by RT with Cobalt-60 or 2-4 megavoltage (MV) photons beam, with local control (LC) rates ranging from approximately 85-94% in T1N0 disease [3-5]. The reported treatment outcome of early GC by primary irradiation with 6 MV photons is limited and conflicting. Some authors reported comparable results with lower energies [6,7] whereas others raised concern about a poorer outcome [8,9]. We present our institution's experience in this report.

## Methods

### Patient characteristics

In mid 2010, we conducted a retrospective analysis of laryngeal cancer patients referred to our center for radical treatment over a 26 year period between January 1983 to December 2005. A total of 1256 consecutive patients were identified. This retrospective study was approved by our Institutional Review Board and Ethics committee. According to the Hong Kong Cancer Registry, about a quarter of all laryngeal cancer cases diagnosed in Hong Kong over that period were treated in our institution. Out of the 1256 patients, there were 433 previously untreated patients with T1N0 GC.

### Staging

All patients had full physical examination, routine blood counts, renal and liver function tests, chest x ray, endoscopic examination and biopsy for histology diagnosis. Computed tomography (CT) scan of larynx and neck was performed in 412 (95%) patients. Patients were restaged according to UICC TNM 2002 classification [10]. Table 1 summarized the various patient, tumor and treatment parameters.

**Table 1:** Patient, tumor and treatment parameters

Parameters	Patients no (%)
Sex	
Male	413 (95.3%)
Female	20 (4.6%)

T stage	
T1a	324 (74.8%)
T1b	109 (25.1%)

Grade	
Well differentiated	154 (35.5%)
Mod differentiated	273 (63.0%)
Poorly differentiated	6 (1.3%)

AC involvement	
Yes	197 (45.4%)
No	236 (54.1%)

Hemoglobin level	
≤ 13 g/dL	45 (10.4%)
> 13 g/dL	388 (89.6%)

Field size (cm2)	
< 30.5	215
30.5-35.5	165
≥ 35.5	53

A. Dose fraction size	
2.5 Gy	177 (40.8)

Total dose (Gy)	
55	30 (6.9)
57.5	134 (30.9)
60	13 (3.0)

Tx duration (days)	
≤ 30	25 (5.7)
31-33	141 (32.5)
≥ 34	11 (2.5)

BEDcGy_15 _(cGy)	
Median	6520
range	6058-6820

B. Dose fraction size	
2.0 Gy	256 (59.1)

Total dose (Gy)	
64	52 (12.0)
66	202 (46.6)
68	2 (0.46)

Tx duration (days)	
≤ 45	48 (11.0)
46-50	203 (46.8)
≥ 51	5 (1.5)

BEDcGy_15 _(cGy)	
Median	6340
range	6040-6700

*Abbreviations*: AC = Anterior Commissure, Tx: treatment,

BEDcGy_15 _: Tumor biologically effective dose

### Radiotherapy Treatment

All patients were treated exclusively with 6-MV photons from linear accelerator (LA). They were treated in a supine position, immobilized with a customized cobex H&N cast. All patients received a continuous course of RT with once-daily fractionation, 5 fractions per week. All fields were equally weighted and treated in each fraction.

### Field size and set up

All patients were treated with parallel-opposed fields, to cover the glottic larynx with 1-2 cm margins. The field size was obtained by multiplying the field length by the field width. It ranged from 22-38.5 cm^2 ^(median: 27.5 cm^2^). Typically, the superior border was put at around the top of the thyroid cartilage, the inferior border at around the bottom of the cricoid cartilage; the anterior border extended beyond the skin surface and the posterior border placed at the anterior edge of vertebral body of the cervical vertebrae. Elective nodal irradiation was not given. Optimized wedge filters were used to improve the dose homogeneity. 0.5 cm thickness wax up bolus was used for diseases involving or close to the anterior commissure (AC). From February 1990, doses were prescribed to the 100% isodose line on a 2- dimensional plan derived from the plane of the patient contour at the level of the isocenter.

### Dose and fractionation

RT dose was prescribed at the midline along the central axis or recalculated at the ICRU reference point. Between the period of 1983-1988 and 1996-2005, patients were treated with a fraction size of 2.0 Gy whereas during 1989-1995, a fraction size of 2.5 Gy was utilized because of constraints in LA machine in our hospital.

We opted to compute the tumor biologically effective dose (BED) by using the standard linear quadratic formula (LQ) with time factors corrected: [11]

where *n *fractions of *d *Gy are given in an overall time of *T *days and kick off time (*T*k) for tumor repopulation. We assume α/β = 15 for laryngeal cancer [12], *T*k = 28 for tumor[13], *T*p = average cell number doubling time during continuing radiation, 3 days for tumor[14]. Alpha (α) = 0.35 Gy^-1 ^[14][coefficient of non-repairable injury, log cell kill (exponentially-based logs) per gray of dose].

One hundred and seventy-seven (40.8%) were treated with a dose fraction size of 2.5 Gy, with total dose of 55-60 Gy (median: 57.5 Gy), within a treatment duration of 30-38 days (median 31 days). The most commonly used dose-fractionation schedule was 57.5 Gy in 23 fractions. Tumor BEDGy_15 _ranged from 60.5 to 68.2 Gy_15 _(median = 65.2 Gy_15_).

Two hundred and fifty- six (59.1%) patients were treated with a dose fraction size of 2.0 Gy, with a total dose of 64-68 Gy (median: 66 Gy), within a treatment duration of 44-58 days (median: 46). The most commonly used dose-fractionation schedule was 66 Gy in 33 fractions. Tumor BEDGy_15 _ranged from 60.4 to 67.0 Gy_15 _(median = 63.4 Gy_15_).

### Follow up and assessment

All patients underwent evaluation of response to treatment by endoscopy examination at 6 to 8 weeks after completion of RT treatment. Patients were regularly seen once every two or three months during the initial 2 years and then six-monthly up to 5 years and then yearly thereafter.

### Complications

Acute and chronic complications were scored according to the Common Terminology Criteria for Adverse Events version 3.0 [15].

### Statistical analysis

Local and neck failure was defined as clinically/radiological detectable disease in larynx and cervical lymph node (LN) respectively. Distant metastasis (DM) was defined as clinically or radiologically detectable disease outside the larynx and cervical LN. Clinicopathologic parameters that were analyzed included age (<61 vs. 61-70 vs. >71), gender (male vs. female), pre-treatment hemoglobin (Hb) level (<13.0 vs. ≥13.0 g/dl), T sub-stage (T1a vs. T1b), tumor grading (well vs. moderate vs. poorly differentiated squamous cell carcinoma), involvement of AC (yes vs. no). Treatment parameters included dose fraction size (2.0 Gy vs. 2.5 Gy), BEDGy_15 _given (< 65.0 Gy_15 _vs. ≥ 65.0 Gy_15_), treatment field size in cm^2 ^(< 30.5 vs. 30.5 - 35.5 vs. > 35.5), and treatment period (1983-1990 vs. 1991-2000 vs. 2001-2005).

All time-related events were measured from date of the first RT treatment. The actuarial local/neck failure rate and ultimate local/neck failure rate were calculated by the Kaplan-Meier method. Difference of the endpoints stratified by the various prognostic factors were evaluated by the Log- rank test.

Cox proportional hazard model was used for both univariate and multivariate analysis to determine the hazard ratios and significance of potential risk factors for local control (LC). All statistical tests were two-sided and performed at the 0.05 level of significance (*p *value). Only factors with a level of significance less than 0.05 in univariate analysis would be further analyzed in the multivariate analysis. We used SPSS, version 15.0, (SPSS Inc., Chicago, IL) for all statistical analyses.

## Results

### Local and Neck control

**Figure 1:**
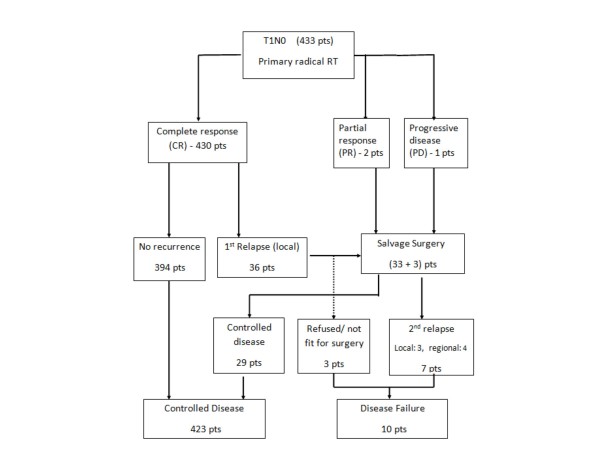
**Clinical Course**. *Abbreviations: *pts = patients; RT = radiotherapy.

**Figure 2:**
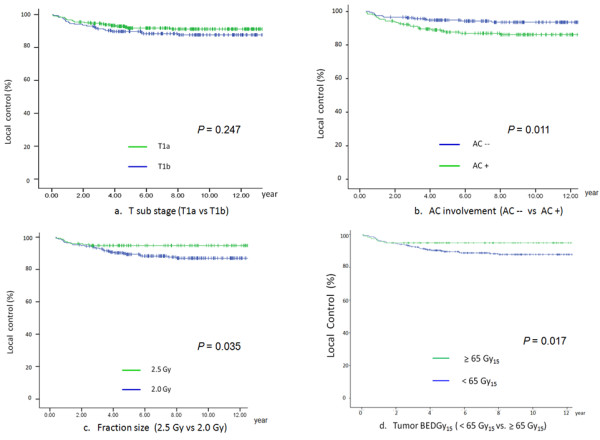
**Local control rate according to T sub-stage; AC involvement; Fraction size; tumor BEDGy**_**15**_. a. T sub stage (T1a vs T1b). b. AC involvement (AC - vs AC +). c. fraction size (2.5 Gy vs 2.0 Gy). d. Tumor BEDGy_15 _(<65 Gy_15 _vs ≧ 65 Gy_15_). *Abbreviations: *AC: anterior commissure; AC--: absence of AC involvement; AC+: presence of AC involvement; BED: biologically effective dose.

The median follow-up time was 10.5 years (range 3.3 - 26.6 years). The clinical course of this patient cohort is shown in figure 1. The 5-year and 10-year LC rates for T1a group were 92% and 91% respectively whereas those for T1b group were 89% and 87% respectively (figure 2a).

Complete response (CR) was achieved in 430 (99.3%) patients, while 3 (0.7%) patients had residual disease/disease progression at vocal cord(s) at 8 weeks after completion of RT. Thirty-six (8.3%) among the 430 patients who achieved CR had their first relapse observed at a median interval of 15 months after completion of RT treatment. All first relapses occurred in the laryngeal glottis and none of them occurred in neck LNs or distant sites.

### Salvage surgery after recurrence/residual disease

Of the 39 patients who developed local recurrence or persistent disease, 36 were salvaged by total laryngectomy. Three patients refused or were not considered medically fit for salvage treatment. Seven patients developed second relapse or progression as regional or distant metastasis despite total laryngectomy, resulting in overall ultimate disease failure in 10 patients. This resulted in an ultimate 10 year LC of 97%. Larynx preservation was achieved in 394 (91%) patients.

### Complications

RT was well tolerated by all patients. No patient had grade III or IV toxicity that necessitated treatment interruption >3 days, nasogastric tube feeding, intravenous fluid supplement or tracheostomy. There is no clinical or radiological chondroradionecrosis that warranted laryngectomy.

### Factors affecting Local Control

On multivariate analysis, LC was adversely affected by poorly differentiated histology (Hazard Ratio [HR]: 7.5, *p *= 0.035); involvement of AC (HR: 2.34, *p *= 0.011); fraction dose size of 2.0 Gy (HR: 2.17, *p *= 0.035) and tumor BEDGy_15 _< 65 Gy_15 _(HR: 3.38, *p *= 0.017) [table 2].

**Table 2:** Univariate and multivariate analysis of factors affecting local control

Parameters	Events/patients	Uni-variate analysis	Multivariate analysis	
		
		*P *value	HR (95% CI)	*P *value
Age				
<61	18/142			
61-70	15/153	0.302	_	_
>70	9/138			

Sex				
Male	41/413	0.445	_	_
Female	1/20			

Sub-stage				
T1A	28/324	0.24	_	_
T1B	14/109			

Grade				
Well diff	9/154		1	
Mod diff	29/273	0.0001*	1.91 (1.2-3.85)	0.035*
Poorly diff	4/6		7.5 (3.42-15.24)	

Hb				
< 13.0	6/45	0.367	_	_
≥ 13.0	36/388			

AC				
No	14/236	0.004*	1	0.011*
Yes	28/197		2.34 (1.21-4.52)	

Field size (cm^2^)				
<30.5	35/215			
30.5-35.5	7/165	0.534	_	_
> 35.5	0/53			

Dose size				
2.0 Gy	32/256	0.021*	2.17 (1.28-4.18)	0.035*
2.5 Gy	10/177		1	

Tumor BED				
< 65 (Gy_15_)	29/239	0.025*	3.38 (1.29-7.83)	0.017*
≥ 65 (Gy_15_)	13/194		1	

Tx period				
1983-1990	10/115			
1991-2000	25/224	0.643	_	_
2001-2005	7/94			

*Abbreviations*: HR = Hazard ratio; CI = confidence interval; Gy = Gray;

diff = differentiated; AC = anterior commissure; BED = Biologically Effective Dose;

* = statistically significant

Figure 2b depicts LC rate according to presence of AC involvement. There was a significant difference in LC between those with presence of AC involvement and without AC involvement (86% vs. 95% at 5 years, 85% vs. 94% at 10 years (*p *= 0.011). Figure 2c depicts LC rate according to fraction size. There was a significant difference between the 2.0 Gy group and the 2.5 Gy group (89% vs. 95% at 5 years; 87% vs. 95% at 10 year, *p *= 0.035). Figure 2d depicts LC rate according to tumor BEDGy_15_. There was a significant difference between the group with tumor BED < 65 Gy_15 _vs. the group with tumor BED ≥ 65 Gy_15 _(90% vs. 96% at 5 years; 88% vs. 96% at 10 years, *p *= 0.017).

We further categorized patients into 4 groups (A1-A4) according to involvement of AC and fraction size (category- A) or another 4 groups (B1-B4) according to involvement of AC and tumor BED (category-B), i.e. (A1) no AC involvement with fraction size of 2.5 Gy, (A2) no AC involvement with fraction size of 2.0 Gy, (A3) presence of AC involvement with fraction size of 2.5 Gy, (A4) presence of AC involvement with fraction size of 2.0 Gy [table 3]; (B1) no AC involvement and BED Gy_15_≥ 65 Gy_15_, (B2) no AC involvement and BED Gy_15_< 65 Gy_15_, (B3) presence of AC involvement and BED Gy_15_≧65 Gy_15_, (B4) presence of AC involvement and BED Gy15 <65 Gy_15 _[table 4].

**Table 3:** Category- A: grouping according to AC involvement and fraction size

	AC-	AC+
2.5 Gy/fraction	94 (A1)	83 (A3)

2.0 Gy/fraction	142 (A2)	114 (A4)

**Table 4:** Category- B: grouping according to AC involvement and BED

	AC-	AC+
BED ≥ 65 Gy_15_	94 (B1)	100 (B3)

BED < 65 Gy_15_	142 (B2)	97 (B4)

*Abbreviations*: AC = anterior commissure; BED = Biologically Effective Dose

**Figure 3:**
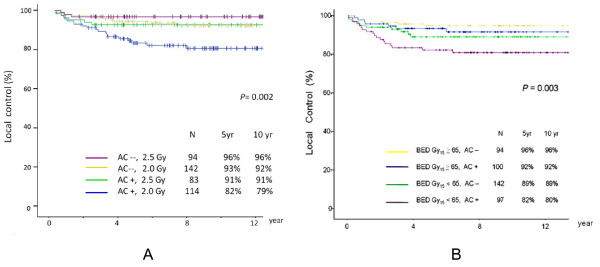
**Local control rate according fraction size, tumor BED 15, AC involvement.** a. fraction size, together with AC involvement. b. tumor BED G15, together with AC involvement. Abbreviations: AC: anterior commissure  tumor BED Gy_15_: tumor biologically effective dose  N: patients numbers  AC-- : absence of AC involvement   AC+: presence of AC involvement .

There was a statistically significant difference in LC rates among 4 groups in category-A: 96% vs. 93% vs. 91% vs. 82% respectively at 5 years; 96% vs. 92% vs. 91% vs.79% respectively at 10 year (*p *= 0.002) [figure 3a]. Again, similar statistically significant difference in LC rates was also observed among 4 groups in category-B: 96% vs. 92% vs. 89% vs.82% at 5 years; 96% vs. 92% vs. 89% vs. 80% respectively at 10 year *p*= 0.003 [figure 3b].

## Discussion

In western countries, both definitive RT and conservative surgery (endoscopic laser surgery/open organ preserving surgery) are accepted standard treatment modalities for stage one GC [16,17]. A survey conducted in eleven regions/countries in Asia revealed that in regions following the 'British school' like Hong Kong and Singapore, RT alone has remained the primary treatment modality for early laryngeal cancers [2]. As laser surgery has become more popular since Stener's landmark report [18], it is expected that it will be increasingly employed in local institutions.

Focusing on primary irradiation, there is extensive literature regarding the efficacy and prognostic factors for RT in early GC [3-5,19-23]. All data except one series [24] was retrospective series. Broadly, prognostic factors can be divided into patient/tumor- as well as treatment- related factors. Apart from stage, other patient or tumor prognostic factors have been reported, including tumor bulk [4,19,25], bilaterality [4,5], AC involvement (see below), tumor grade [3,26] and hemoglobin level [5,26,27]. Radiation treatment- related factors included dose fraction size, total dose, overall treatment time (OTT) [see below].

The majority of these published data were derived from patients treated by Cobalt-60 machine or LA generating 2-4 MV photons [3-5,21,26]. In many RT centers, these therapy units have been decommissioned. With a general shift from the use of Cobalt-60 to LA treatment units, it is anticipated that 6 MV photon beams generated by LA will become the prevailing workhorse for treatment in clinical practice [28]. Table 5 showed published results for T1N0 GC treated with 6 MV photons in the recent two decades.

**Table 5:** Reports in literature on results of T1N0 glottic cancer treated with 6 MV photons

Author year [ref]	Patients no	Total Dose (Gy)	Dose size (Gy)	Local Control (5 year)%
Akine et al. 1991 [7]	151	62.5-67.5	2.0-2.4	89

Fein et al. 1996 [27]	43	66	2	95

Foote et al. 1996 [6]	27	63	2.25	100

Lee et al. 2001 [28]	86	66	2	T1a: 82 T1b: 76

Gowda et al. 2003 [36]	100	50-52.5	3.12-3.28	T1a: 93 T1b: 89

Franchin et al. 2003 [20]	323	63-65.2	2.25	T1: 90

Sjögren et al. 2009 [37]	59	60	2.0-2.8	T1a: 87 T1b: 85

current study	433	57.5-66	2.0-2.5	T1a: 92 T1b: 89

The impact of AC involvement on the RT treatment outcome of early GC is still controversial. The so called AC or Broyle's tendon is the insertion of vocalis tendon into thyroid cartilage in the area of AC. This is considered as a weak point for tumor spread because in this area, there is no thyroid cartilage perichondrium to resist tumor spread. Although some data suggested that AC involvement portended a worse prognosis, it has not been included in the staging system.

In the recent two decades, many authors identified AC involvement as one of the independent poor prognostic factors in LC for T1N0 GC treated by primary RT [4,21,29]. In a recent report by Smee et al. [30], it was found that AC involvement was one of the independent poor prognostic factors for LC as well as cause specific survival. One explanation is related to the possibility of 'understaging' without CT scan staging, as patients might have a larger tumor burden anteriorly, and in some cases unrecognized subglottic extension [31]. In our patient cohort, since 95% of patients had evaluation by CT scan, the issue of under-staging should be minimal.

Another probable reason is the theoretical risk of under-dosage at the air- tissue interface with the depth-dose characteristics of 6 MV photons compared with those of Cobalt-60 beam. This is related to inadequate tissue present at the area of AC where the neck is thin, as well as lack of electronic equilibrium at the air-tissue interface which might be more pronounced with high-energy photons treated with small field size [32,33]. Hence, poorer coverage of the prescribed dose to the tumor may occur in early glottic tumors with AC involvement, particularly when treated with 6 MV photons. Sombeck et al. [34] performed a dosimetric evaluation comparing 6MV photons with Cobalt-60 beam. They revealed that there was no significant difference in the dose received at any point along the vocal cords. On the other hand, a recent study by Spirydovich [35] demonstrated a significant under- dosage occurring at the air-tissue interface of larynx treated by 6 MV photons. The authors performed Monte Carlo dose calculation to CT-based mathematical neck. They identified that at least 5% of a hypothetical tumor of 3.5 cm^3 ^received less than 86% of the maximum tumor dose in neck that contains air cavities in comparison to 91% of the maximum tumor dose in the homogeneous neck.

However, some other major reports did not reveal the impact of AC on LC of early glottic cancer [3,5,36,37].

With regard to the impact of dose fraction size for early glottic disease, there is little controversy that inferior LC is associated with fraction size < 2.0 Gy when patients are treated once daily, 5 days per week [38,39].

Among the reports published in the literature, the common contemporary irradiation schedules for T1N0 GC included: 66 Gy in 33 fractions in 6.5 weeks, 63 Gy in 28 fractions in 5.5 weeks, and 60 Gy in 25 fractions in 5 weeks [17,40]. In fact, a prospective randomized study from Yamazaki et al. [24] demonstrated a statistically superior 5-year LC rate of 92% for patients treated with fraction size of 2.25 Gy compared with 77% for those treated with 2.0 Gy.

Besides, many reports have shown that prolonging OTT in T1N0 GC has an adverse impact on LC and dose compensation is needed to maintain the tumor control probability. Indeed, several authors have highlighted the complex inter- relationship among the variables of total dose, fraction size and OTT [41,42].

Fowler [43] commented that according to radiobiological principles, even if there would be a positive effect of increasing total dose or fraction size on LC, and a strong negative effect of treatment prolongation, these effects become minimal where the LC was already at a very high level, because of the plateau of the slope of the sigmoid- shaped dose-response curve above 70 or 80%. This theoretical postulation has also been verified by observations reported. Fein et al.[27] and Le et al. [21] did not observe a relationship between fraction size and LC. Although there was a trend for higher LC in patients treated with fraction size of ~2.25 Gy when compared to smaller fraction size, the difference did not reach statistical significance. The authors attributed the lack of difference to the low recurrence rate in T1 lesions, thus under- powering the studies to demonstrate a significant relationship between fraction size and LC.

The debate over these discrepancies was rebuffed after the impact of shortening of OTT in LC of H&N cancers was confirmed in randomized trials with accelerated schedules. Both the Danish Head and Neck Cancer Study Group study (DAHANCA 6 & 7) [44] and the International Atomic Energy Agency (IAEA- ACC) trial [45] delivered six fractions per week but keeping same total dose, enabled a treatment of 66 Gy in 33 fractions to be given in 8 days less than the conventional schedule. They revealed a 10-12% improvement in LC of H&N cancers (especially for early laryngeal cancer subset) upon shortened OTT. It appeared that by shortening the OTT, treatment outcome is improved as accelerated repopulation of tumor clonogens would be reduced. But these accelerated schedules are also shown to have more acute radiation toxicity in terms of severe skin reactions, confluent mucositis necessitating tube feeding.

In evaluating the efficacy of various fractionation schedules, we opted to test the impact of tumor BEDGy_15 _which incorporates the components of fraction size, OTT and total dose. Our analysis shows that tumor BED ≥ 65 Gy_15 _is associated with better LC. Table 6 illustrated the common radiation schedules in which fraction size is > 2.0 Gy, the resulting tumor BEDGy_15 _would be > 65 Gy_15 _but the BEDs for both early mucosa and late normal tissues are well below the corresponding dose constraints for complications [aim at 59-63 Gy_10 _for acute mucosa; < 117 Gy_3 _for late normal tissue respectively] [46].

**Table 6:** Calculated tumor BED (Gy_15_), acute mucosal BED (Gy_10_) and late normal tissue BED (Gy_3_) for common radiation schedules

Dose size (Gy)	Fraction number	Total dose (Gy)	Overall treatment time (OTT) in days	Tumor BED (Gy_15_)	**Acute Mucosal BED (Gy_10_) (aim 59-63 Gy_10_) **[46]	**Late normal BED (Gy_3_) (aim <117 Gy_3_) **[46]	references
2.0	33	66	45	64.60	49.1	110	[22,25,27] & current study

2.25	28	63	38	66.45	52.62	110.2	[3,5,20,24,37]

2.5	23	57.5	31	65.28	52.87	105.4	current study

BED = biologically effective dose

= total dose (1 + fraction size/[α/β]) - log_e _2 (*OTT *-*T*k)/α*T*p [14]

Assume: 1. α/β = 15 for laryngeal cancer [12]

2. *T*k: kick off time (*T*k) for tumor repopulation = 28 days [13]

3. *T*p = average cell number doubling time during continuing radiation, 3 days for tumor [14]

4. Alpha (α) = [coefficient of non-repairable injury, log cell kill (exponentially-based logs) per gray of dose]

= 0.35 Gy^-1 ^(14)]

Since the treatment field size for T1N0 GC is small, it permits slight hypofractionated schedule without causing excessive acute radiation toxicity. Shortened OTT overcomes the accelerated repopulation of tumor clonogens.

This also supports the current contemporary practice of fraction dose size > 2.0 Gy (i.e. 2.25 Gy) for treatment of T1N0 GC by other centers [3,6,20,21,24,37]

To the best of our knowledge, our report is the largest study on RT outcomes in T1N0 GC primarily treated with 6 MV photons. As the treatment of choice for early GC in our institution or Hong Kong at large has been and in the near future will still be RT alone [2], this represents a relatively unselected cohort of patients. While this study spans a considerable period of time, the clinical evaluation and treatment techniques have been consistent over the years, thus allowing a valid analysis to be performed. Our results demonstrate that the LC rate with primary RT with 6 MV photons is comparable and agrees with other reports of "unremarkable" treatment outcome difference when comparing Cobalt-60 beam and 6 MV photons [3,5-7,27].

### However, we observe that AC involvement is associated with a poor LC rate

We suspect that the issue of 'cold spot' is more apparent at the AC region, especially when treated with 6MV photons. Certainly, further dosimetric evaluation is needed to validate this suspicion. While involvement of AC is an adverse prognostic factor, we have shown that its negative impact can be overcome by delivering a higher tumor BED (≧ 65 Gy_15_). In order to achieve this tumor BED level in conventional schedule of five daily fractionation each week, we recommend that fraction size > 2.0 Gy should be utilized. In fact, modest hypofractionation is safe and effective for T1N0 GC in terms of both LC and morbidity. Having a shorter OTT is more convenient for patients and is also more cost- effective for RT facility implication.

Nevertheless, the results need to be interpreted with caution, because the current report was a retrospective, single institution study and therefore subjected to biases. For example, we did not have volume measurements on tumor, which has been shown in other reports as one of the important prognostic factors in LC [4,19,25]. In fact, AC involvement may reflect "tumor bulk" and thus may represent a surrogate marker for tumor volume. We suggest the degree of AC involvement should be further defined to better evaluate and confirm its significance in outcome prognostication. We also agree with some authors that the degree of AC involvement should be incorporated into the new UICC staging system for better comparison of results among various studies [47]. Besides, modification of the RT treatment technique like adding anterior field/anterior oblique field can be considered to combat under-dosage at AC [3,20].

## Conclusions

Our data concur with other published result about the efficacy of RT with 6 MV photons for T1N0 GC. While involvement of AC is associated with poor LC rate, its negative impact could be overcome by delivering a higher tumor BED through using fraction size of >2.0 Gy. We recommend that fraction size > 2.0 Gy should be utilized, for radiation schedules with five daily fractions each week.

## Competing interests

The authors declare that they have no competing interests.

## Authors' contributions

CCT participated in the study's design and coordination, performed acquisition of data and drafted the manuscript. KHA and FYC participated in data analysis and revised the manuscript. RKCN and SMC participated in study's design and revised the manuscript. JSKA, YTF and SCKL revised manuscript critically for important intellectual content. All authors read and approved the final manuscript.
